# Factors influencing medical students’ choice of emergency medicine as a career specialty—a descriptive study of Saudi medical students

**DOI:** 10.1186/s12245-018-0174-y

**Published:** 2018-03-07

**Authors:** Hadeel Alkhaneen, Faisal Alhusain, Khalid Alshahri, Nawfal Al Jerian

**Affiliations:** 1 College of Medicine, King Saud bin Abdulaziz University for Health Sciences, Riyadh, Saudi Arabia; 2 Department of Emergency Medicine, Ministry of National guard health affairs, P.O Box 86871, Riyadh, 11632 Saudi Arabia

**Keywords:** Emergency medicine, Medical education, Future career, Saudi Arabia

## Abstract

**Background:**

Choosing a medical specialty is a poorly understood process. Although studies conducted around the world have attempted to identify the factors that affect medical students’ choice of specialty, data is scarce on the factors that influence the choice of specialty of Saudi Arabian medical students, in particular those planning a career in emergency medicine (EM). In this study, we investigated whether Saudi medical students choosing EM are influenced by different factors to those choosing other specialties.

**Methods:**

A cross-sectional survey was conducted at King Saud bin Abdulaziz University for Health Sciences (KSAUHS), Riyadh, Saudi Arabia. The questionnaire distributed among all undergraduate and postgraduate medical students of both sexes in the second and third phases (57% were males and 43% were females).

**Results:**

A total of 436 students answered the questionnaire, a response rate of 53.4%. EM group was most influenced by hospital orientation and lifestyle and least influenced by social orientation and prestige provided by their specialty. Unlike controllable lifestyle (CL) group and primary care (PC) group, EM reported lesser influence of social orientation on their career choice. When compared with students primarily interested in the surgical subspecialties (SS), EM group were less likely to report prestige as an important influence. Moreover, students interested in SS reported a leaser influence of medical lifestyle in comparison to EM group. When compared with CL group, EM group reported more interest in medical lifestyle.

**Conclusions:**

We found that students primarily interested in EM had different values and career expectations to other specialty groups. The trends in specialty choice should be appraised to meet future needs.

## Background

Choosing a medical specialty is a complex, poorly understood process with implications for both the student and physician workforces. It is an important predictor of the future supply of doctors in different specialties. Identifying the factors underlying the choice of a specialty may improve our understanding of students’ preferences for particular specialties and help careers advisors to provide better guidance for medical students [1, 2]. Factors that influence the choice of a medical specialty include the following: specialty-related lifestyle, exposure to a role model, income, length of residency training, varied scope of practice, and patient population. Recently, “controllable lifestyle” (CL) became a significant factor when choosing a specialty [2]. A study undertaken by Dorsey et al. [2] found that 55% of the variation in the specialty preferences of United States (US) medical graduates between 1996 and 2002 could be explained by their CL [2]. Specialties that can provide a CL, such as dermatology, anesthesiology, and emergency medicine (EM), are attracting increasing numbers of medical students every year [1, 3].

Studies conducted around the world, including in the USA [4], Canada [1, 5], Brazil [6], and Japan [7], have attempted to identify the factors that affect medical students’ choice of specialty. However, the process by which medical students in the Middle East choose their future career requires further investigation. A study undertaken at Kuwait University showed that observing good treatment outcomes followed by working in a demanding specialty are the most influential factors when choosing a specialty according to Kuwaiti medical students [8]. Factors associated with choosing a career in primary care (PC) have been well-studied in an attempt to address the decreasing number of students interested in this field [1, 9]. However, few studies have endeavored to determine the factors associated with choosing a career in EM [1, 7]. One study involving Canadian medical students primarily interested in a career in EM between 2001 and 2004 found that medical lifestyle, hospital orientation, and varied scope of practice play a major role in their career choice [1].

EM is considered a new medical specialty and is expanding at a phenomenal pace worldwide. EM training in Saudi Arabia was established in the year 2000 by newly graduated physicians who had completed their EM training in the USA. The first batch of Saudi Arabian students to graduate from the Saudi Board of Emergency Medicine (SBEM) in 2004 consisted of four physicians. The number of available residency positions in the specialty has increased over time. In 2012, 80 qualified EM physicians graduated from the SBEM [10]. However, data is scarce on the factors that affect the choice of specialty of Saudi Arabian medical students, in particular those planning a career in EM. Therefore, in this study, we investigated whether Saudi Arabian medical students choosing EM are subjected to different influential factors to students choosing other specialties.

## Methods

### Study design

This cross-sectional study was conducted at the King Saud bin Abdulaziz University for Health Sciences (KSAU-HS), Riyadh, Saudi Arabia, from March to May of the 2016/2017 academic year. The aim of this study was to determine whether students who are interested in a career in EM have a different profile of influential factors to students choosing other specialties.

Specialties were classified into four groups: EM, PC, CL, and surgical specialty (SS). CL specialties were determined based on a study by Schwartz et al. [3], which examined the influence of CL on specialty choice. The CL group consisted of anesthesiology, neurology, ophthalmology, radiology, otolaryngology, pathology, dermatology, and psychiatry. The PC group included internal medicine, family medicine, pediatrics, and obstetrics/gynecology. The SS group included general surgery, plastic surgery, urology, and orthopedic surgery. Although Schwartz et al. [3] considered EM to be a CL specialty, EM was excluded from the CL Group because it was our group of interest.

### Study setting and population

This study was conducted in the College of Medicine at the KSAU-HS. It is the first public university in the Kingdom of Saudi Arabia and Middle East region specializing in health sciences. Its headquarters is in the main campus in Riyadh, Saudi Arabia. It has two other campuses in Jeddah and Al-Ahsa. The KSAU-HS provides both undergraduate and postgraduate programs: a 6-year program for undergraduates and a 5-year program for postgraduates. The college uses a problem-based learning medical education curriculum that is split into three phases: pre-med, pre-clinical, and clinical. Each phase is 2 years long. Students are termed medical students after completing the 2-year pre-med phase.

### Questionnaire

We distributed a 41-item self-administered and validated questionnaire to all undergraduate and postgraduate medical students of both sexes in the second and third phases, i.e., first- to fourth-year students, and excluded first-phase, pre-med students. The questionnaire was adapted from a study by Scott et al. [1] and used with permission. The questionnaire was carefully revised so that it fulfilled the objectives of the study. The survey gave students a choice between eight major career options: EM, family medicine, internal medicine, obstetrics/gynecology, pediatrics, psychiatry, surgery, and “other” (a write-in choice). They were asked to indicate with a “yes” or “no” which they considered a possible career and to rank their top three career choices. The students then evaluated the extent to which 27 variables influenced their first choice, using a 5-point Likert scale ranging from 1 (no influence) to 5 (major influence). Demographic data were also collected.

### Data management and analyses

Demographic characteristics, including sex, academic year, and specialty preference, were summarized and reported in terms of frequencies and percentages. Factors that influenced students’ career choices were categorized into six new descriptive groups (medical lifestyle, social orientation, prestige, hospital orientation, role model, and varied scope of practice) according to the ability of items to fit in these categories. Table 1 shows the categorization. Social orientation of medicine includes the desire to contribute to the community, patient population and the ability to have enough free time to spend with family and friends. Hospital orientation focuses more on the practice inside the hospital and what kind of care are going to be provided (urgent vs non-urgent). Prestige mainly means income and status among medical profession. Medical lifestyle was defined by Schwartz et al. [3] as “control of work hours”. Factors related to the impact of role model and variety of practice was also considered. The mean ± standard deviation of the Likert scale data for each unique factor and for the new generated categories among career interest groups were calculated. Analysis of variance with a Bonferroni post hoc test was used to compare the influential factors among the different career interest groups. A *P* value ≤ 0.05 was considered to indicate statistical significance. All computations were performed using SPSS version 23 (IBM Corp., Armonk, NY, USA).

**Table 1:** Factors and underlying influences that affect the career choices of medical students

Medical lifestyle	Acceptable on-call schedule
	Research interest
	Acceptable hours of practice
	Flexibility inside of medicine
	Flexibility outside of medicine
	Keep options open
	Short postgraduate training
	Less intense residency program
Social orientation	Patient population is interesting
	Focus on patients in community
	Long-term patient relationship
	Social commitment
	Health promotion is important
	Able to spend appropriate time with my family
Prestige	High income potential
	Status among colleagues
	Stable/secure future
Hospital orientation	Focus on in-hospital care
	Focus on urgent care
	Focus on non-urgent care
	Intervention results immediate
	Don’t like uncertainty
	Prefer medical to social problems
Role model	Good match to career
	Emulate physician
	Meaningful past experience
	Experiences in health fields during medical school
	Experiences with role models during medical school
Varied scope of practice	Wide variety of patient problems
	Narrower variety of patient problems

For each factor, the average of the Likert scores for items in that factor was computed. Analysis of variance was then used to compare factor scores according to career choice

## Results

A total of 436 students answered the questionnaire, a response rate of 53.4%. Of the respondents, 57% were males and 43% were females. PC specialties were the most commonly cited first-choice of our students, followed by SS specialties. Only 7% of the students named EM as their first-choice specialty. Twenty-eight students expressed no specific preference regarding their future career specialty and were excluded from the study. Table 2 shows the baseline characteristics and specialty groups of the participants.

**Table 2:** Baseline characteristics and specialty groups (*n* = 436)

Variable	Category	*n*	%
Sex	Male	250	57
	Female	186	43
Academic year	First	131	30
	Second	134	31
	Third	118	27
	Final	53	12
Specialty category	EM	32	7
	CL	80	18
	PC	163	37
	SS	133	31
	No preference	28	6

*EM* emergency medicine, *CL* controllable lifestyle, *PC* primary care, *SS* surgical specialty

Hospital orientation, social orientation, and medical lifestyle factors were the most frequently cited factors influencing the process of choosing a medical career (with high influence, *P* <  0.001). Hospital orientation factors were the most commonly cited factors in the EM and SS groups. Prestige was the least commonly reported factor in the EM group and one of the top three influential factors in the SS group. Social orientation factors were the most commonly cited factors in the PC and CL groups. The effect of a role model was the least commonly reported factor in the PC and CL groups. Interestingly, varied scope of practice had the least impact on the EM group compared with the other three groups. Table 3 shows a comparison of factors influencing the career interests of medical students.

**Table 3:** Comparison of factors that influence the career interests of medical students

Factors	% of the students				Results of ANOVA	
	EM *n* = 32	CL *n* = 80	PC *n* = 163	SS *n* = 133	*F*	*P* value
Medical lifestyle	3.24	3.25	3.22	2.9	7.9	< 0.001
Social orientation	3.14	3.39	3.48	3.12	8.81	< 0.001
Prestige	2.8	3.05	2.93	3.13	2.27	0.08
Hospital orientation	3.45	3.13	3.26	3.5	7.87	< 0.001
Role model	3.14	2.68	2.94	3	2.94	0.033
Varied scope of practice	3.19	3.28	3.25	3.24	0.12	0.948

*ANOVA* analysis of variance, *EM* emergency medicine, *CL* controllable lifestyle, *PC* primary care, *SS* surgical specialty

Students choosing EM reported that its focus on urgent care and high-income potential were the most important influences on their career choice. CL specialties were chosen for their acceptable hours of practice and mostly acceptable on-call schedule. For those who chose a PC specialty, the patient population and health promotion were considered important influences. Observing an immediate result in their patients was the most influential factor cited by students in the SS group. The variables that influenced students’ career interests upon entry to medical school are presented in Table 4.

**Table 4:** Variables that influence students’ career interests at medical school

Factor	% of the students				Results of ANOVA	
	EM *n* = 32	CL *n* = 80	PC *n* = 163	SS *n* = 133	*F*	*P* value
Wide variety of patient problems	3.97	3.15	3.27	3.35	3.96	0.008
Narrower variety of patient problems	2.41	3.4	3.22	3.13	4.88	0.002
Good match to career	2.75	2.55	2.55	2.38	0.86	0.464
Patient population is interesting	4.13	3.9	3.94	3.95	0.36	0.783
Focus on in-hospital care	3.41	3.45	3.63	3.8	2.4	0.067
Focus on patients in community	2.87	3.4	3.61	3.12	6.53	< 0.001
Focus on urgent care	4.25	2.63	2.9	3.74	26.5	< 0.001
Focus on non-urgent care	2.47	3.56	3.39	3.03	10.2	< 0.001
Intervention results immediate	4.22	2.99	3.32	3.98	18.84	< 0.001
High income potential	3.13	3.55	3.27	3.72	5.35	0.001
Long-term patient relationship	2.28	3.02	3.16	2.73	6.16	< 0.001
Status among colleagues	2.97	3.07	3.17	3.19	0.44	0.726
Acceptable on-call schedule	3.31	3.68	3.28	2.94	6.38	< 0.001
Do not like uncertainty	2.81	3.09	3.04	3.04	0.44	0.728
Prefer medical to social problems	3.53	3.04	3.26	3.44	2.08	0.102
Emulate physician	3.03	2.64	3.09	3.28	3.56	0.014
Research interest	3.03	3.59	3.26	3.28	1.82	0.143
Social commitment	2.84	2.86	2.98	2.65	1.73	0.16
Stable/secure future	2.31	2.54	2.36	2.48	0.56	0.644
Health promotion is important	3.06	3.68	3.67	3.28	5.25	0.001
Acceptable hours of practice	3.66	3.81	3.52	3.1	7.54	< 0.001
Flexibility inside of medicine	3.69	3.62	3.64	3.26	4.06	0.007
Flexibility outside of medicine	3.44	3.51	3.28	2.96	4.49	0.004
Keep options open	3.53	3.19	3.59	3.17	4.76	0.003
Meaningful past experience	3.5	2.71	2.96	3.11	3.21	0.023
Short postgraduate training	2.75	2.26	2.68	2.24	3.9	0.009
Less intense residency program	2.53	2.36	2.53	2.23	1.54	0.204
Experiences in health fields during medical school	3.13	2.78	3.02	3.1	1.1	0.35
Experiences with role models during medical school	3.31	2.74	3.07	3.14	2.53	0.057
Able to spend appropriate time with my family	3.63	3.45	3.5	2.98	6.06	< 0.001

*ANOVA* analysis of variance, *EM* emergency medicine, *CL* controllable lifestyle, *PC* primary care, *SS* surgical specialty

We studied specialty choices in relation to the baseline characteristics of the respondents, as depicted in Table 5. Interestingly, male students were more numerous than females in the surgical specialties (69%). Moreover, the sexes were more balanced in the EM group, with only slightly more male students (56%). Females tended to choose CL and PC specialties more often than male students. Of great interest was the finding that students’ preferences for surgical specialties decreased with years of study, whereas that for PC specialties increased. The rank order of factors influencing specialty choice for all four specialty groups was determined based on the mean for each factor, as shown in Table 6. Hospital orientation and medical lifestyle were the two most influential factors in the EM group. Conversely, the EM group was least influenced by prestige and social orientation. Varied scope of practice was among the top three influential factors in all groups. Meanwhile, prestige was among the bottom three factors affecting the EM, CL, and PC groups, but not the SS group.

**Table 5:** Comparison of students’ demographic characteristics according to career choice

Demographic characteristics	% of students (*n* = 436)			
	EM	CL	PC	SS
Men compared with women	56	44	51	69
Men’s preferences	8	15	36	40
Women’s preferences	8	25	44	23
First-year students’ preferences	9	16	38	38
Second-year students’ preferences	7	24	39	31
Third-year students’ preferences	11	16	42	32
Final-year students’ preferences	2	27	44	27

*EM* emergency medicine, *CL* controllable lifestyle, *PC* primary care, *SS* surgical specialty

**Table 6:** Rank order of factors by specialty group

Rank	EM	CL	PC	SS
1	Hospital orientation	Social orientation	Social orientation	Hospital orientation
2	Medical lifestyle	Varied scope of practice	Hospital orientation	Varied scope of practice
3	Varied scope of practice	Medical lifestyle	Varied scope of practice	Prestige
4	Role model	Hospital orientation	Medical lifestyle	Social orientation
5	Social orientation	Prestige	Role model	Role model
6	Prestige	Role model	Prestige	Medical lifestyle

*EM* emergency medicine, *CL* controllable lifestyle, *PC* primary care, *SS* surgical specialty

## Discussion

In recent years, the nature of medical specialization in Saudi Arabia has undergone many changes. Recently, a comprehensive plan to reform the economy and health, education, infrastructure, recreation, and tourism sectors of Saudi Arabia, termed Saudi Vision 2030, was unveiled. This plan will improve the integration and continuity of health service provision. Therefore, an increase in residency positions in PC and EM can be expected in the future [11]. Choosing a medical specialty is an important step that impacts the health-care workforce. It is an important predictor of the future supply of physicians in different specialties. According to Zeldow et al. [12] and DeWitt et al. [13], 45–70% of medical students enter the specialty they preferred during medical school after they graduate. Thus, EM educators must be encouraged to understand the attitudes and priorities of medical students choosing a career in EM, the promotion of which may help to maximize the number of EM applicants. In this study, we aimed to investigate the influence of different factors on choosing a specialty.

Our findings revealed that only 7% of the medical students surveyed considered EM their first-choice specialty. This is consistent with other studies performed in the USA and Canada, which reported that EM was the first-choice specialty of 10 and 6.1% of students, respectively. We also found that 33.2% of the students considered EM a possible career and ranked it among their top three career choices.

Our study also provides insight into the factors that influence the career choices of medical students. Our data suggest that hospital orientation and medical lifestyle are the most influential factors when choosing EM as a future specialty. A focus on urgent care and observing immediate results of an intervention were the most frequently reported factors in the EM group, followed by the SS group. Conversely, a focus on non-urgent care was the most frequently cited factor in the CL and PC groups. In addition, we found that students who were interested in EM were more concerned about the medical lifestyle provided by their specialty than students who chose a CL specialty. According to Dorsey et al. [2], because of the similarities between EM and CL specialties, EM can be considered a CL specialty. The term CL was first introduced in 1989 by Schwartz et al. [3] and defined as the ability to have control over working hours.

However, in comparison to other CL specialties, the lifestyle provided by EM as a medical specialty differs. The flexibility of working hours is an advantage of EM. In our study, flexibility of working hours as an influence on career choice was most frequently cited in the EM group compared with the other groups. The EM group also reported the strong influence of the intensity and length of the training program. The lifestyle provided by EM may explain the high career satisfaction reported by EM physicians. In a longitudinal study of career satisfaction associated with EM, 65.2% of EM physicians reported high career satisfaction [14]. However, of all the specialties, EM physicians tend to have the highest rates of burnout. A recent study found that at least half of EM physicians suffered burnout as early as the second year of training in an EM residency program [15].

The effect of exposure to a role model on medical students can be summarized in three main outcomes: shaping of career aspirations, development of a professional identity, and behavior [15]. We found that the influence of mentors and role models on the choice of medical specialty was low in all four groups. It is difficult to compare our studies to studies conducted in the USA because of differences in the curriculum and teaching methods. However, exposure to a role model was ranked as the second most influential factor on medical students’ choice of a career in EM and the first most influential factor for their choice of other specialties in US medical colleges [4]. Moreover, salary expectation was considered more of influence for American medical students who are interested in EM [4]. These differences between students could be explained by the cultural and social status in each country, which could affect the different abovementioned domains. A lack of formal career counseling for our students may be the reason. A trial of a mentoring program was established in the northeastern USA in an attempt to increase the number of students interested in PC. First-year medical students who were interested in PC were paired with PC mentors. Those students were successfully matched to PC programs at a rate of 87.5%. They appreciated the relationship with their mentors, which positively influenced their interest in a PC career [16]. In our study, the effect of mentors and role models was ranked as the fourth most influential factor for students interested in a career in EM. However, of the students surveyed, the EM group reported the greatest influence of exposure to role models in their desired health field during medical school. This result may be explained by the excellent EM Department at the National Guard Hospital where our students receive a compulsory 4-week training course after their first year at medical college.

EM educators are in short supply compared with more established specialties such as surgery and internal medicine [17]. Establishing meaningful mentoring relationships between EM physicians and students may help to provide future mentors, because some of these students will develop into future leaders and mentors in the field of EM [17].

Perceived prestige and financial reward were most commonly associated with a surgical career choice. The SS group reported the highest influence of salary and status among colleagues. In contrast to the SS group, prestige had the least influence on the career choices of the EM Group.

## Conclusions

The trends in specialty choice should be appraised to meet the future needs of the Kingdom of Saudi Arabia. We found that students primarily interested in EM have different values and career expectations to those interested in careers in other specialties. We showed that hospital orientation and medical lifestyle are influential factors on the formulation of their specialty preferences. These findings may help program directors and chairs in the specialty of EM to attract more students. Our findings may also be helpful to medical students and careers counselors in the specialty selection process. We cannot make a prediction about the EM health workforce in Saudi Arabia based on our results. However, we can conclude that students who are interested in EM are concerned about the hospital orientation and lifestyle that the training program in EM provides.

## Abbreviations

CL: Controllable lifestyle
EM: Emergency Medicine
KSAU-HS: King Saud bin Abdulaziz University for Health Sciences
PC: Primary Care
SBEM: Saudi Board of Emergency Medicine
SS: Surgical Subspecialty
US: United States
