# Correction to: Factors influencing medical students’ choice of emergency medicine as a career specialty—a descriptive study of Saudi medical students

**DOI:** 10.1186/s12245-018-0216-5

**Published:** 2018-12-17

**Authors:** Hadeel Alkhaneen, Faisal Alhusain, Khalid Alshehri, Nawfal Al Jerian

**Affiliations:** 1 College of Medicine, King Saud bin Abdulaziz University for Health Sciences, Riyadh, Saudi Arabia; 2 Department of Emergency Medicine, Ministry of National guard health affairs, P.O Box 86871, Riyadh, 11632 Saudi Arabia

## Correction

Following publication of the original article [1], the authors reported that one of the authors’ names is spelled incorrectly. In this Correction the incorrect and correct author name are shown. The original publication of this article has been corrected.

Originally the author name has been published as:

- Khalid Alshahri

The correct author name is:

- Khalid Alshehri
